# Dissecting the Mechanisms of Doxorubicin and Oxidative Stress-Induced Cytotoxicity: The Involvement of Actin Cytoskeleton and ROCK1

**DOI:** 10.1371/journal.pone.0131763

**Published:** 2015-07-02

**Authors:** Lei Wei, Michelle Surma, Gina Gough, Stephanie Shi, Nathan Lambert-Cheatham, Jiang Chang, Jianjian Shi

**Affiliations:** 1 Riley Heart Research Center, Herman B Wells Center for Pediatric Research, Department of Pediatrics, Indiana University, School of Medicine, Indianapolis, Indiana, United States of America; 2 Texas A&M University Health Science Center, Institute of Biosciences and Technology, Houston, Texas, United States of America; University of Sheffield, UNITED KINGDOM

## Abstract

We have recently reported that ROCK1 deficiency in mouse embryonic fibroblasts (MEF) has superior anti-apoptotic and pro-survival effects than antioxidants against doxorubicin, a chemotherapeutic drug. Although oxidative stress is the most widely accepted mechanism, our studies suggest that ROCK1-dependent actin cytoskeleton remodeling plays a more important role in mediating doxorubicin cytotoxicity on MEFs. To further explore the contributions of ROCK1-dependent actin cytoskeleton remodeling in response to stress, this study investigates the mechanistic differences between the cytotoxic effects of doxorubicin *versus* hydrogen peroxide (H_2_O_2_), with a focus on cytoskeleton alterations, apoptosis and necrosis induction. We found that both types of stress induce caspase activation but with different temporal patterns and magnitudes in MEFs: H_2_O_2_ induces the maximal levels (2 to 4-fold) of activation of caspases 3, 8, and 9 within 4 h, while doxorubicin induces much higher maximal levels (15 to 25-fold) of caspases activation at later time points (16–24 h). In addition, necrosis induced by H_2_O_2_ reaches maximal levels within 4 h while doxorubicin-induced necrosis largely occurs at 16–24 h secondary to apoptosis. Moreover, both types of stress induce actin cytoskeleton remodeling but with different characteristics: H_2_O_2_ induces disruption of stress fibers associated with cytosolic translocation of phosphorylated myosin light chain (p-MLC) from stress fibers, while doxorubicin induces cortical F-actin formation associated with cortical translocation of p-MLC from central stress fibers. Furthermore, N-acetylcysteine (an antioxidant) is a potent suppressor for H_2_O_2_-induced cytotoxic effects including caspase activation, necrosis, and cell detachment, but shows a much reduced inhibition on doxorubicin-induced changes. On the other hand, ROCK1 deficiency is a more potent suppressor for the cytotoxic effects induced by doxorubicin than by H_2_O_2_. These results support the notion that doxorubicin induces caspase activation, necrosis, and actin cytoskeleton alterations largely through ROCK1-dependent and oxidative stress-independent pathways.

## Introduction

The undesirable toxicity of chemotherapeutic agents to normal tissues affects their therapeutic efficiency. Doxorubicin, a good example, is used to treat a wide spectrum of hematologic malignancies and solid tumors. However, the dose of doxorubicin needs to be closely monitored as it can cause life-threatening cardiotoxicity [1–5]. The mechanisms of doxorubicin-induced cytotoxicity to normal cells have been under intense investigation for many years [4–13]. Reactive oxygen species (ROS) generated by doxorubicin has been the most studied cause of cardiotoxicity, and is believed to act as a major trigger for several forms of cell death including apoptosis, necrosis, and autophagy [4–17]. However, clinical trials of antioxidant therapy showed insufficient beneficial effects [18,19], and the reasons for this under-expected outcome are still unclear.

In addition to generating free radicals, doxorubicin also affects actin cytoskeleton stability via inhibition of actin polymerization [20,21]. We have recently reported that ROCK1 plays an important role in stress fiber disassembly induced by doxorubicin leading to impaired cell adhesion and apoptosis in mouse embryonic fibroblasts (MEFs) [22,23]. At the molecular level, we observed that ROCK1 increases myosin light chain (MLC) phosphorylation and peripheral actomyosin contraction [22,23]. ROCK is the central regulator of the actin cytoskeleton downstream of small GTPase RhoA [24–33]. The two ROCKs, ROCK1 and ROCK2, encoded by distinct genes, are highly homologous with an overall amino acid sequence identity of 65% [24–26]. Our recent studies reveal that ROCK1 deficiency (but not ROCK2 deficiency) in MEFs not only exhibits greater protective effects than antioxidants, but also significantly increases the beneficial effects of antioxidants against doxorubicin-induced cytotoxicity including apoptosis and cell detachment [34]. These studies suggest that ROCK1-dependent actin cytoskeleton remodeling plays a more important role than ROS generation in mediating doxorubicin cytotoxicity, at least in MEFs.

To further explore the contribution of actin cytoskeleton alterations to doxorubicin-induced cytotoxicity, this study compares the cytotoxic effects induced by doxorubicin *versus* those induced by hydrogen peroxide (H_2_O_2_), one of the most frequently used oxidative stresses in cell biology. We found that both H_2_O_2_ and doxorubicin induce caspase activation, necrosis, actin cytoskeleton remodeling, and increased intracellular ROS levels in MEFs but with significantly different characteristics. Furthermore, N-acetylcysteine (NAC), an antioxidant, is a more potent suppressor for H_2_O_2_-induced than doxorubicin-induced cytotoxic effects, while ROCK1 deficiency has more potent inhibitory effects on doxorubicin-induced than H_2_O_2_-induced cytotoxicity. These results support the notion that doxorubicin induces actin cytoskeleton alterations, caspase activations, and necrosis largely through ROS-independent and ROCK1-dependent pathways.

## Results

### H_2_O_2_ and doxorubicin induce caspase activation with different temporal patterns and magnitudes in MEFs

**Fig 1:**
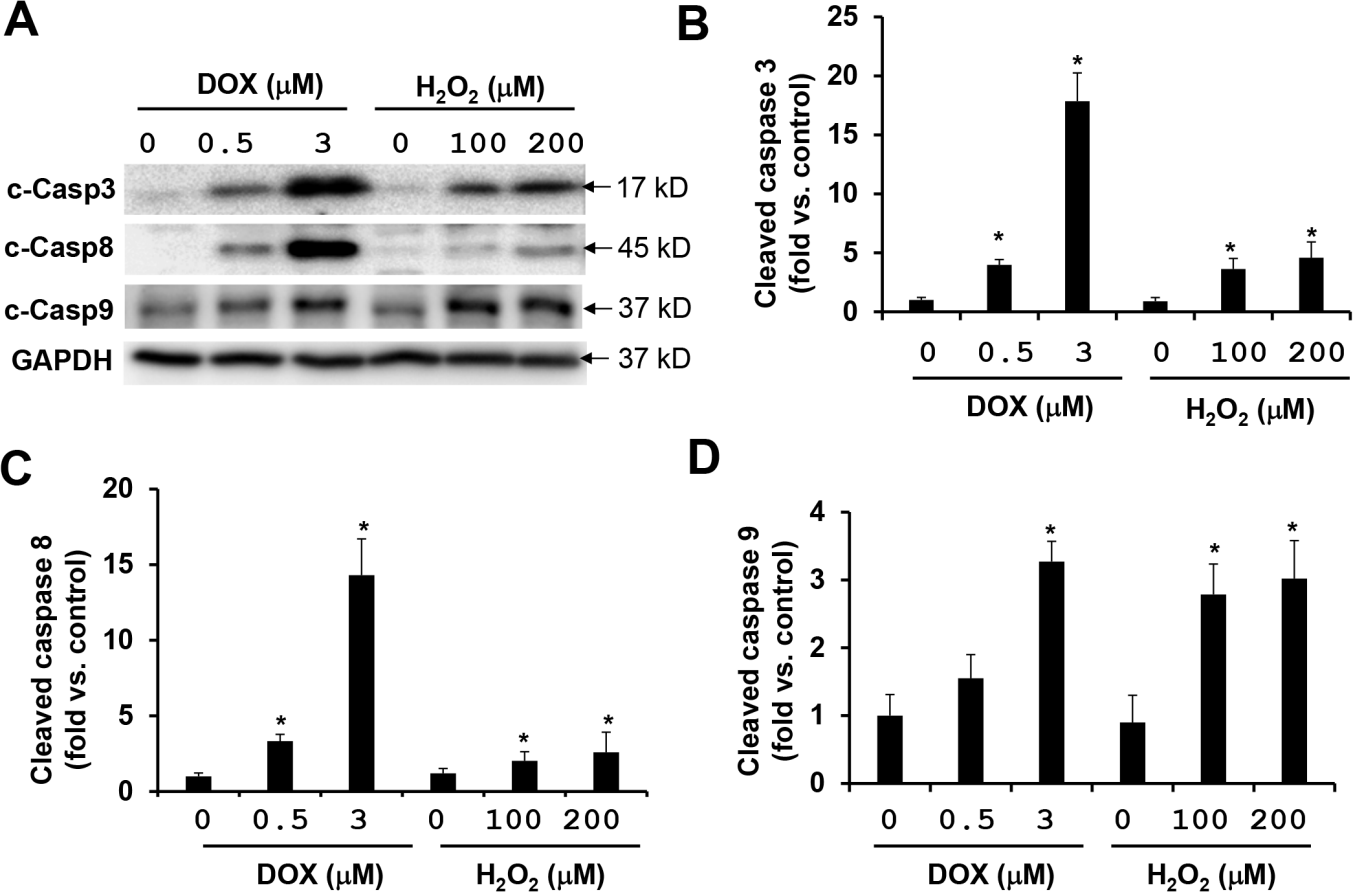
Doxorubicin is more potent than H_2_O_2_ at inducing caspase activation. (A)Representative image showing a Western blot of cleaved caspases 3, 8, and 9 in cell lysates from attached WT MEFs treated with H_**2**_O_**2**_ or doxorubicin at indicated concentrations for 16 h. Equal amount of proteins were loaded. (B-D) Quantitative analysis of immunoreactive bands of cleaved caspases 3, 8, and 9 expressed as fold change relative to WT control. n = 4–6 for each condition. * P < 0.05 vs. control.

It is believed that ROS generation induced by doxorubicin plays an important role in caspase activations, and the caspases serve as the primary mediators of apoptosis. Cleaved caspase 3 is a central marker for the activation of the caspase cascades, which are the results of the activation of either extrinsic pathway involving caspase 8, or intrinsic mitochondrial pathway involving caspase 9. We previously observed that antioxidants NAC and diphenyleneiodonium (an inhibitor of NADPH oxidase) inhibit doxorubicin-induced activations of caspases 3, 8, and 9 by only 20–30% in MEFs while ROCK1 deficiency inhibits 70–80% of them [34]. This suggests that ROS-independent pathways play a predominant role in mediating the pro-apoptotic effects of doxorubicin. To further test this hypothesis, we compared the effects of H_2_O_2_ and doxorubicin on caspase activations in MEFs. In a dose-dependent study, we observed that the increased activation levels (2 to 4-fold) of caspase 3 and caspase 8 induced by 100 or 200 μM of H_2_O_2_ were significantly lower than those (15 to 25-fold) induced by 3 μM of doxorubicin after 16 h of treatment (Fig 1A–1C). Caspase 9 activation levels were comparable for these two inducers (Fig 1A and 1D). In addition, neither increasing nor decreasing the concentrations of H_2_O_2_ could further change the activation level of caspases 3, 8, and 9 (Fig A in S1 Fig) indicating that the H_2_O_2_ concentration ranges of 100 to 200 μM generate near maximum for caspase activations in MEFs. On the other hand, increasing doxorubicin up to 5–10 μM can further increase caspase 3, 8, and 9 activation (Fig B in S1 Fig).

**Fig 2:**
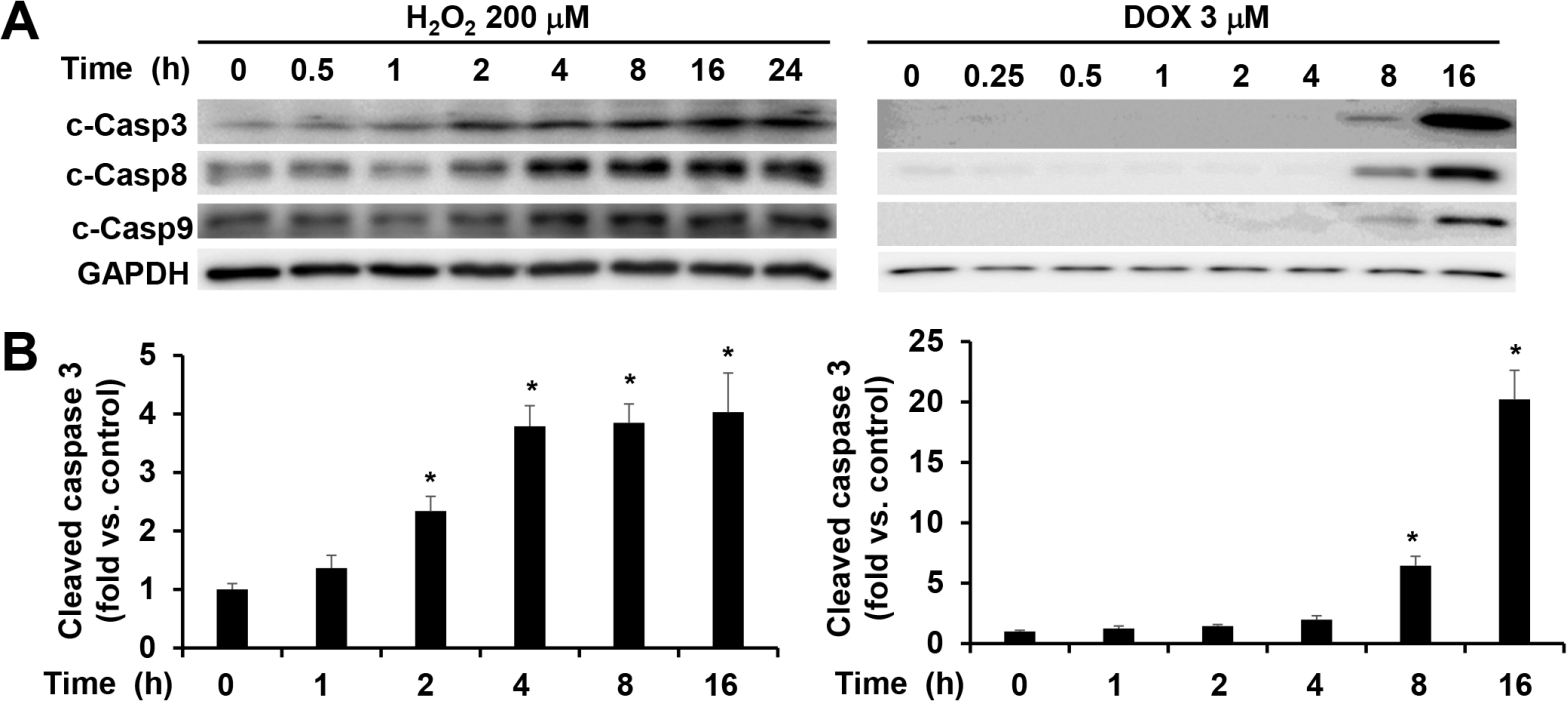
Different time courses of caspase activation by H_2_O_2_ versus doxorubicin. (A) Representative image of Western blot of cleaved caspases 3, 8, and 9 in cell lysates from attached WT MEFs treated with 200 μM of H_**2**_O_**2**_ or 3 μM of doxorubicin at different time points as indicated. Equal amount of proteins were loaded. (B) Quantitative analysis of immunoreactive bands of cleaved caspase 3 expressed as fold change relative to WT control. n = 4–6 for each condition. * *P* < 0.05 vs. control.

In addition to the different levels of activations, the temporal patterns of the caspase activations were also different for H_2_O_2_ and doxorubicin treatments. The maximal levels of activation of caspases 3, 8, and 9 induced by 200 μM of H_2_O_2_ were reached within 4 h and no further increases were observed up to 24 h of treatment (Fig 2). In comparison, the activation of these caspases was detectable at 8 h after receiving 3 μM of doxorubicin treatment and continuously increased till reaching a plateau at 16 h (Fig 2, data at 24 h were similar to those at 16 h, S2 Fig). These results indicate that doxorubicin is a more potent inducer of caspase activation than H_2_O_2_, especially for caspase 3 and caspase 8.

It is worth noting that the time window for doxorubicin to induce caspase activation (8 to 24 h) in WT MEF cells (Figs 1 and 2) is consistent with our previous observation in neonatal mouse ventricles which also showed a marked increase of caspase 3 activation at 8 h after doxorubicin injection [35].

### NAC is a potent suppressor for H_2_O_2_-induced caspase activations, but has limited effect on doxorubicin-induced caspase activation

**Fig 3:**
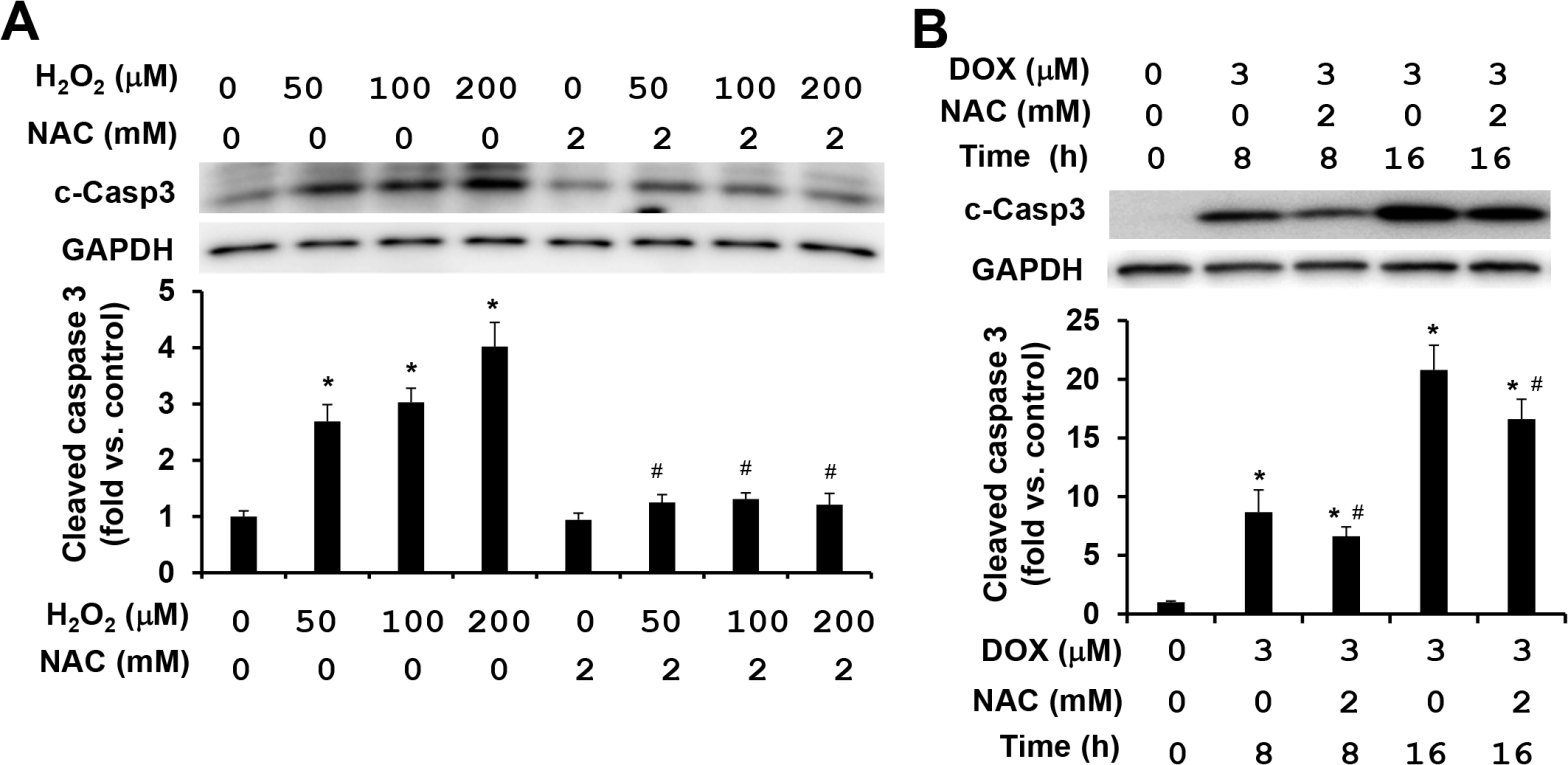
NAC attenuates H_2_O_2_-induced caspase activation, but shows limited effect on doxorubicin-induced caspase activation. **(**A) Representative image (top) and quantitative analysis (bottom) of Western blot of cleaved caspase 3 in cell lysates from attached WT MEFs treated with increasing concentrations of H_**2**_O_**2**_ and/or 2 mM NAC for 4 h. Equal amount of proteins was loaded. (B) Representative image (top) and quantitative analysis (bottom) of Western blot of cleaved caspase 3 in cell lysates from attached WT MEFs treated with 3 μM doxorubicin and/or 2 mM NAC for 8 or 16 h. n = 4–6 for each condition. * *P* < 0.05 vs. control. ^#^ *P* < 0.05 vs. H_**2**_O_**2**_ or doxorubicin only condition.

NAC, an analog and precursor of glutathione, has been used in the clinical practice for many decades with well characterized molecular mechanisms [36,37]. Because the time needed reaching the plateau of caspases 3, 8, and 9 activations was different under H_2_O_2_ and doxorubicin treatments (Fig 2), we assessed the effects of NAC on caspase activations in MEFs after 4 h of H_2_O_2_ treatment and 8 or 16 h of doxorubicin treatment. As expected, the treatment with 2 mM of NAC completely suppressed H_2_O_2_-induced caspase activations (Fig 3A). In contrast, the treatment with the same concentration of NAC exhibited only a partial reduction of less than 30% on doxorubicin-induced caspase 3 activation (Fig 3B). In addition to the treatment mentioned above, increasing NAC concentrations from 2 to 10 mM, or decreasing doxorubicin concentrations from 3 to 1 μM, the limited effects of NAC on doxorubicin-induced caspase activation were still persistent (<30%) (S3 Fig). These results indicate that doxorubicin-induced caspase activations are largely mediated by ROS-independent mechanisms.

### H_2_O_2_ and doxorubicin induce actin cytoskeleton remodeling with different characteristics

**Fig 4:**
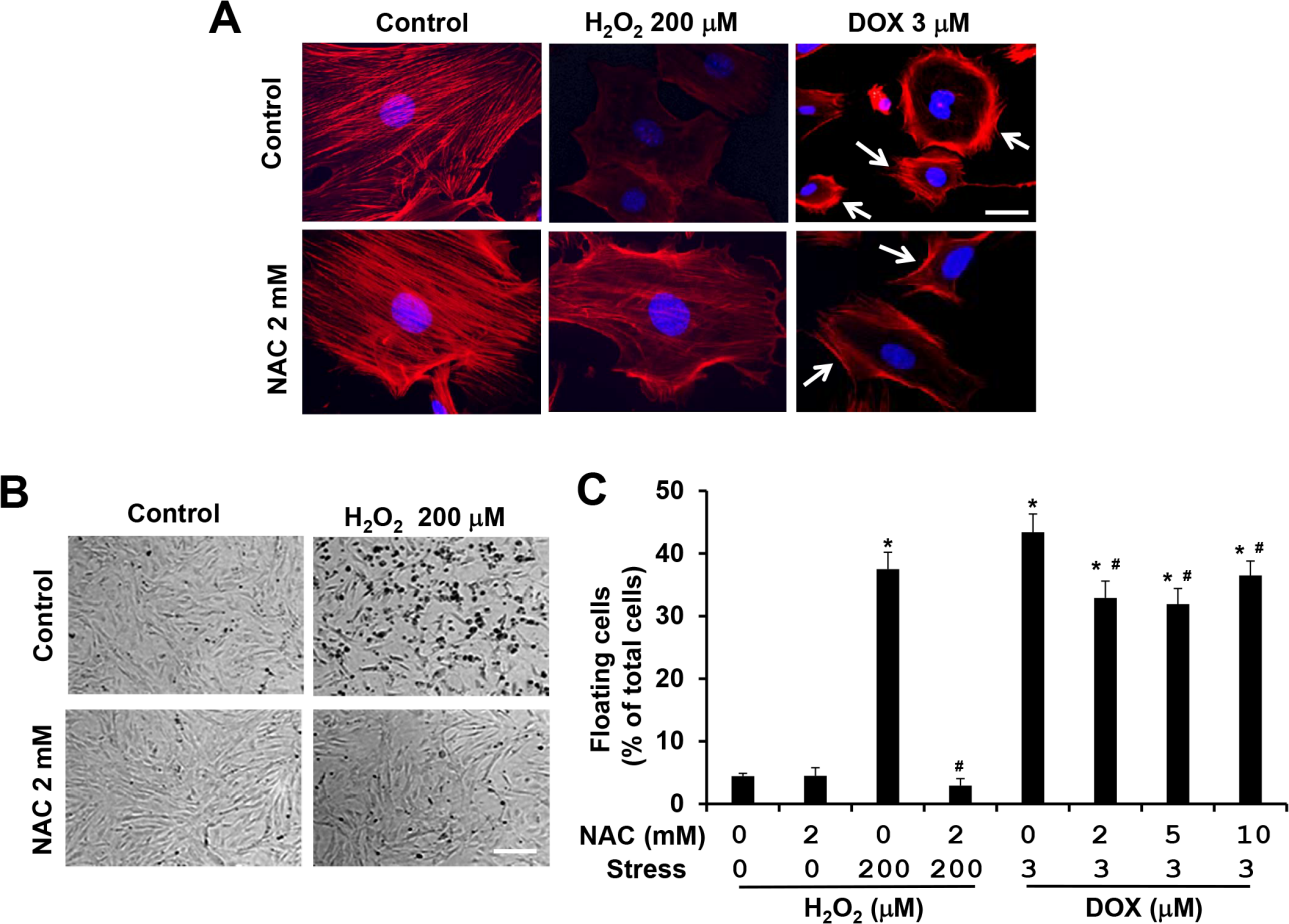
NAC attenuates H_2_O_2_-induced disruption of actin cytoskeleton and cell detachment, but shows limited effect on doxorubicin-induced actin cytoskeleton alteration and cell detachment. (A) Representative images of rhodamine-phalloidin staining for F-actin (red) and DAPI for nucleus (blue) of WT MEFs treated with 200 μM of H_**2**_O_**2**_ and/or 2 mM NAC for 4 h or with 3 μM of doxorubicin and/or 2 mM NAC for 8 h. Cells showing enriched cortical actin cytoskeleton are indicated with white arrows. Bar, 25 μm. (B) Representative images of bright field photography of WT MEFs treated with 200 μM of H_**2**_O_**2**_ and/or 2 mM NAC for 16 h showing H_**2**_O_**2**_-induced cell detachment. Bar, 400 μm. (C) Both floating and attached cells were collected after 16 h treatment with 200 μM of H_**2**_O_**2**_ and/or 2 mM NAC. Both floating and attached cells were also collected after 16 h treatment with 3 μM doxorubicin and/or increasing concentration of NAC as indicated. Floating cell ratio was expressed as percentage of total cells (floating plus attached cells) under each treatment condition. * *P* < 0.05 vs. control. ^#^ *P* < 0.05 vs. H_**2**_O_**2**_ or doxorubicin only condition.

Our previous studies revealed that doxorubicin induces actin cytoskeleton remodeling with the formation of a cortical contractile ring at the cell periphery and the disruption of central stress fibers, resulting in impaired cell adhesion and increased cell detachment [22,23] (Fig 4A and 4C). Different from doxorubicin-induced actin cytoskeleton alterations, treatment with H_2_O_2_ at 200 μM for 4 h did not induce cortical actin formation; instead, stress fiber formation was homogenously reduced (Fig 4A). Consistent with this finding, H_2_O_2_ treatment impaired cell adhesion and increased cell detachment (Fig 4B and 4C). Moreover, co-treatment with 2 mM of NAC completely suppressed the disruption of actin cytoskeleton and cell detachment induced by 200 μM of H_2_O_2_ (Fig 4A–4C). On the other hand, co-treatment with 2 mM of NAC and 3 μM of doxorubicin did not suppress the disruption of central stress fibers, but slightly reduced the formation of cortical actin (Fig 4A) resulting in partial inhibition (<30%) of cell detachment (Fig 4C). These results indicate that H_2_O_2_ and doxorubicin induce actin cytoskeleton alterations through different mechanisms in MEFs.

### ROCK1 deletion is a potent suppressor for doxorubicin-induced actin cytoskeleton remodeling and cell detachment, but shows minor effects on H_2_O_2_-induced cell detachment

**Fig 5:**
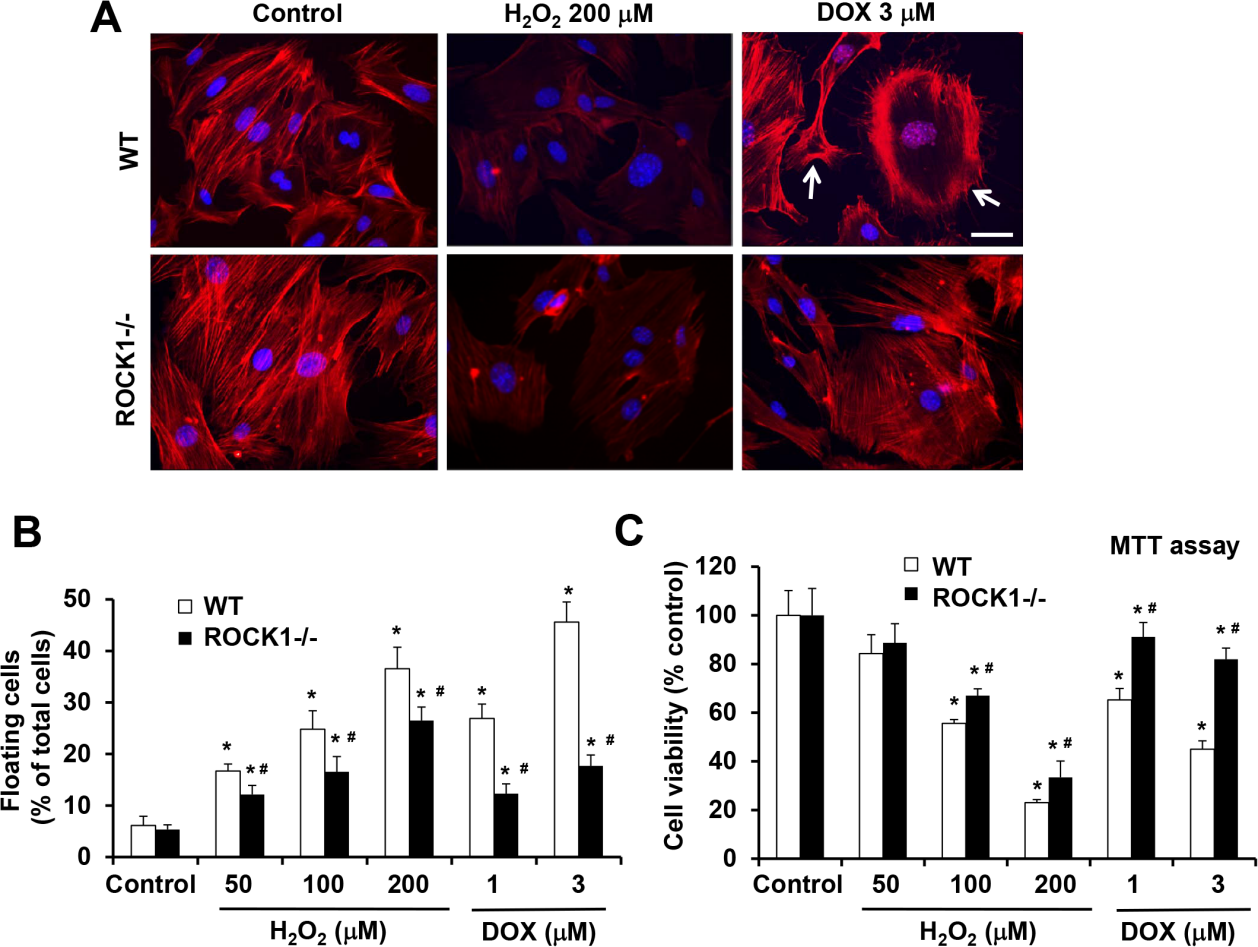
ROCK1 deletion has limited effects on H_2_O_2_-induced disruption of actin cytoskeleton, cell detachment and cell death. (A) Representative images of stained F-actin (red) and nucleus (blue) in WT and *ROCK1* ^*-/-*^ MEFs treated with 200 μM of H_**2**_O_**2**_ for 4 h or with 3 μM of doxorubicin for 8 h. Cells showing enriched cortical actin cytoskeleton are indicated with white arrows. Bar, 25 μm. (B) Both floating and attached WT and *ROCK1* ^*-/-*^ MEFs were collected after 16 h treatment with increasing concentrations of H_**2**_O_**2**_ as indicated. Both floating and attached cells were also collected after 16 h treatment with 1 or 3 μM doxorubicin. Floating cell ratio was expressed as percentage of total cells (floating plus attached cells) under each treatment condition. (C) MTT assay was performed on WT and *ROCK1* ^*-/-*^ MEFs treated with increasing concentrations of H_**2**_O_**2**_ or with 1–3 μM doxorubicin for 16 h. Cell viability was expressed as percentage of control cells without treatment. * *P* < 0.05 vs. control of the same genotype. ^#^ *P* < 0.05 vs. WT under the same treatment condition.

We have revealed that the deletion of ROCK1 in MEFs reduces doxorubicin-induced actin cytoskeleton alterations and cell detachment, and significantly improved cell viability [22,23] (Fig 5A–5C). To examine the impact of ROCK1 deficiency on H_2_O_2_-induced actin cytoskeleton remodeling, we performed F-actin staining and cell detachment assays in WT and *ROCK1* ^*-/-*^ MEFs after H_2_O_2_ treatment at the concentrations of 100, 200, and 300 μM, and at different time points. The ROCK1 deletion had insignificant effects on H_2_O_2_-induced actin cytoskeleton disruption. Fig 5A shows representative images observed in WT and *ROCK1* ^*-/-*^ MEFs treated with 200 μM H_2_O_2_ for 4 h. Comparing the 16 h of treatment effects between H_2_O_2_ and doxorubicin, *ROCK1* ^*-/-*^ MEFs demonstrated about 25% reduction in cell detachment induced by 200 μM H_2_O_2_, and about a 60% reduction induced by 3 μM doxorubicin (Fig 5B). Consistently, ROCK1 deletion had a smaller impact on cell viability in H_2_O_2_ treatment than in doxorubicin treatment as measured by 3-(4,5-dimethylthiazol-2-yl)-2,5-diphenyltetrazolium bromide (MTT) assay (Fig 5C). These results reveal that ROCK1 deficiency provides considerably more effective protection from doxorubicin-induced cytotoxicity than H_2_O_2_-induced cytotoxicity.

**Fig 6:**
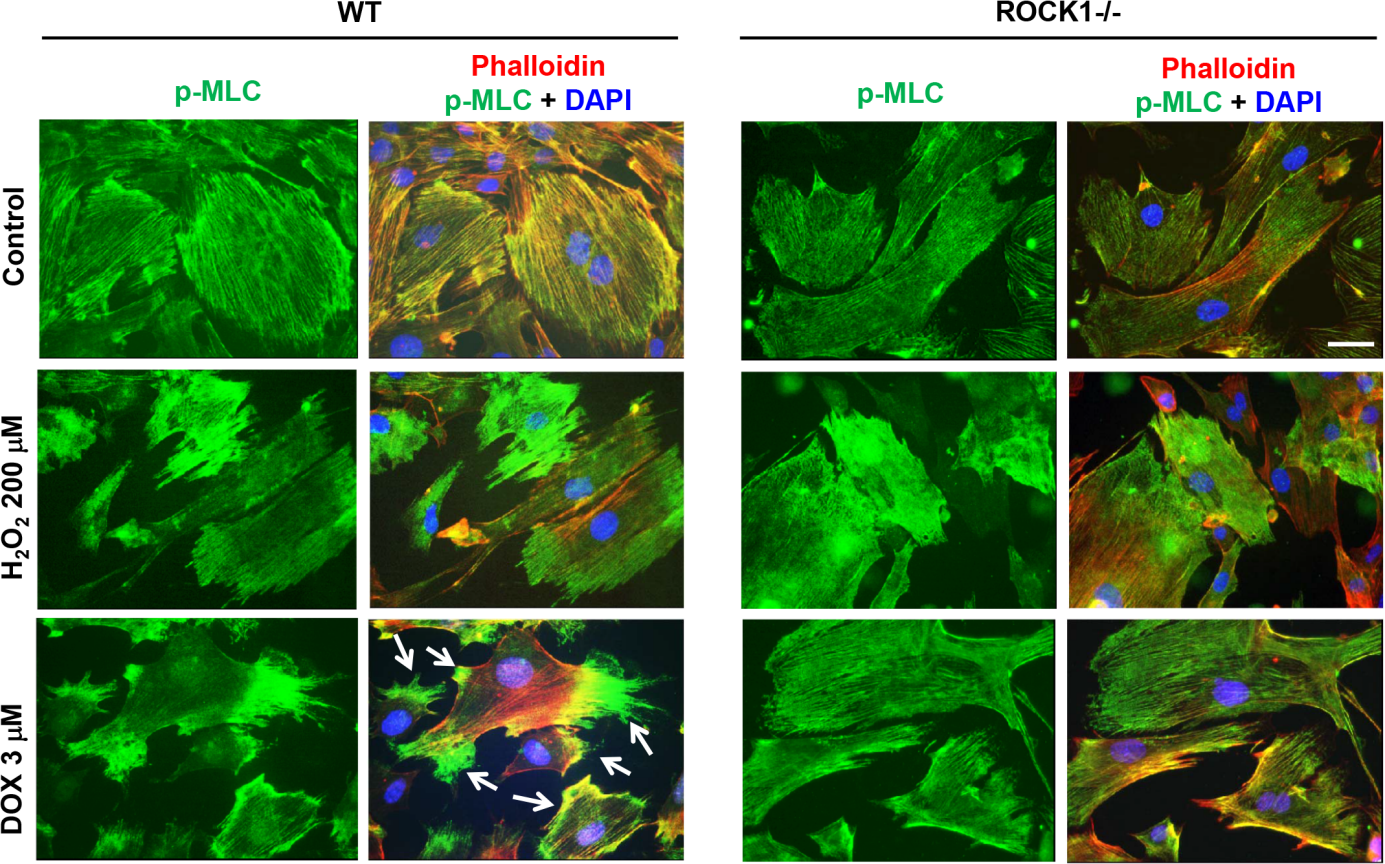
H_2_O_2_ and doxorubicin treatments show different alteration on MLC phosphorylation. Representative images of F-actin staining (red), p-MLC (green), and nucleus (blue) in WT and *ROCK1* ^*-/-*^ MEFs treated with 200 μM of H_**2**_O_**2**_ for 4 h or with 3 μM of doxorubicin for 8 h. Cells showing enriched cortical p-MLC staining are indicated with white arrows. Bar, 25 μm. Doxorubicin induces an increase in p-MLC translocation to cortical actin cytoskeleton in WT MEFs but to a much smaller extent in *ROCK1* ^*-/-*^ MEFs. WT and *ROCK1* ^*-/-*^ MEFs treated with H_**2**_O_**2**_ both show diffused cytoplasmic p-MLC staining.

Phosphorylated MLC2 is a critical component of stress fibers and co-localized with F-actin on stress fibers under control conditions in *ROCK1* ^*-/-*^ and WT MEFs (Fig 6). To further examine the effects of H_2_O_2_ and doxorubicin on actin cytoskeleton remodeling, we performed immunochemistry to stain p-MLC2 in WT and *ROCK1* ^*-/-*^ MEFs which were treated with 3 μM of doxorubicin or 200 μM of H_2_O_2_. We found that in WT MEFs, doxorubicin and H_2_O_2_ had different impacts on p-MLC2 staining in MEFs: in doxorubicin treatment, p-MLC2 was reduced in the central stress fibers and translocated to the thick stress fibers on the cell periphery, but in H_2_O_2_ treatment, p-MLC2 was dissociated from both central and cortical stress fibers, and translocated to the cytosol (Fig 6). Compared with WT MEFs, ROCK1 deficient MEFs showed reduced translocation of p-MLC2 from central stress fibers to cortical stress fibers after doxorubicin treatment, however similar translocation of p-MLC2 from stress fibers to the cytoplasm in H_2_O_2_ treatment occurred in ROCK1 deficient MEFs (Fig 6).

After a short time period of H_2_O_2_ treatment (from 2 to 4 h), p-MLC was heterogeneously located in the cytosol, the pattern could be high or low in individual MEF (Fig 6). However when treatment lasted from 6 to 8 h, the pattern became homogenously low (S4 Fig). On the other hand, cortical translocation of p-MLC in WT MEFs after doxorubicin treatment was most noticeable at 8 h before the peak of cortical actin ring formation at 16 h. In addition, co-treatment with 1 μM of blebbistatin, a direct inhibitor of myosin II ATPase, inhibited doxorubicin-induced cell detachment as previously reported [22], however it increased H_2_O_2_-induced cell detachment (S5 Fig). Together, these results indicate that doxorubicin and H_2_O_2_ induce different subcellular translocation of p-MLC resulting in different actin cytoskeleton alterations: Increased cortical actomyosin contraction, which is ROCK1 dependent, contributes to doxorubicin-induced cell detachment; while reduced actomyosin contraction and disruption of the actin cytoskeleton, which is independent of ROCK1, contributes to H_2_O_2_-induced cell detachment.

### ROCK1 deletion is a potent suppressor for doxorubicin-induced apoptosis and necrosis, and only inhibits H_2_O_2_-induced necrosis but not apoptosis

**Fig 7:**
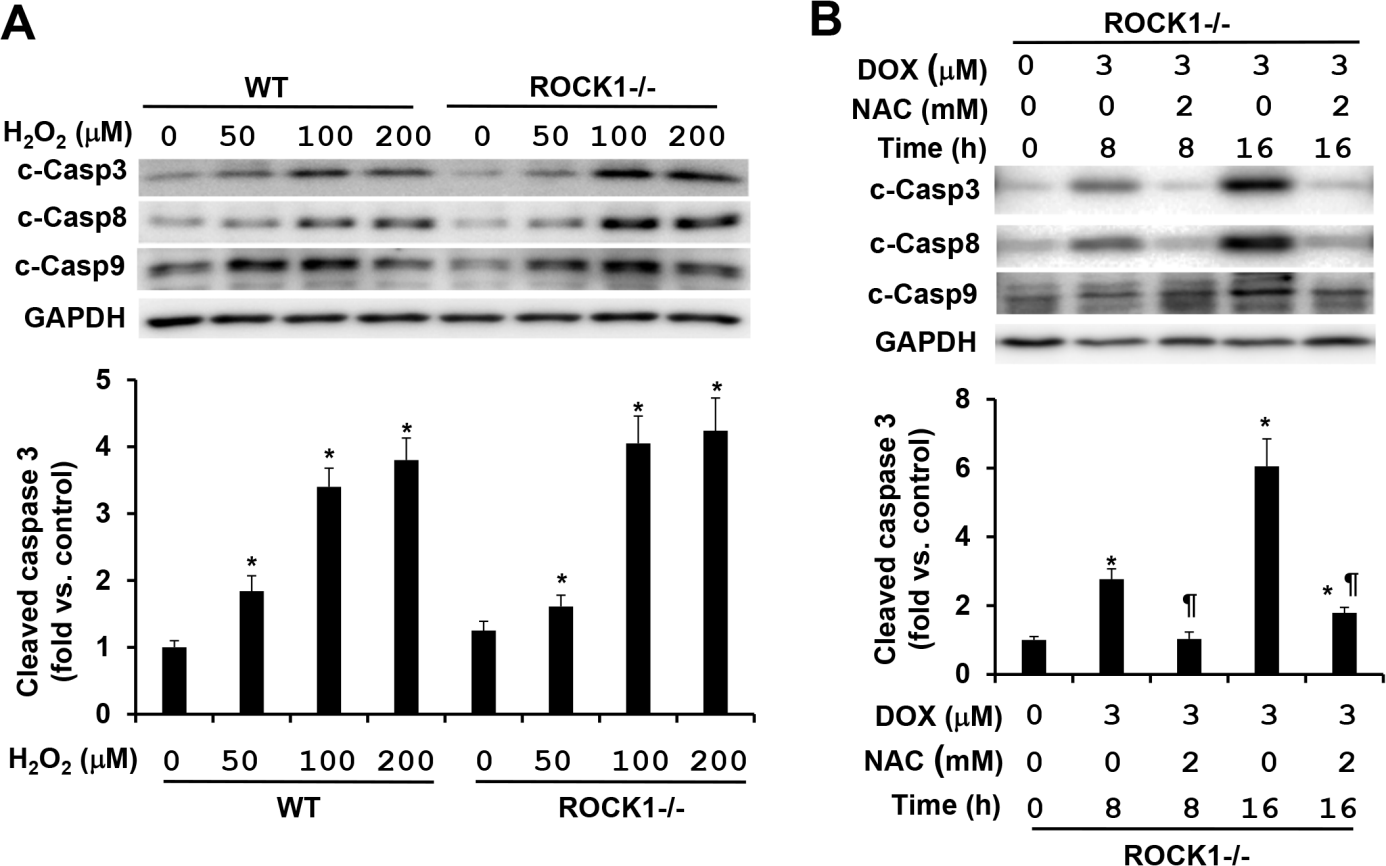
ROCK1 deletion has no inhibition on H_2_O_2_-induced caspase activation. (A). Representative image of Western blot of cleaved caspases 3, 8, and 9 (top) and quantitative analysis (bottom) of Western blot of cleaved caspase 3 in cell lysates from attached WT and *ROCK1* ^*-/-*^ MEFs treated with increasing concentrations of H_**2**_O_**2**_ for 4 h. Equal amount of proteins was loaded. (B) Representative image of Western blot of cleaved caspases 3, 8, and 9 (top) and quantitative analysis (bottom) of Western blot of cleaved caspase 3 in cell lysates from attached *ROCK1* ^*-/-*^ MEFs treated with 3 μM doxorubicin and/or 2 mM NAC for 8 or 16 h. Similar experiments performed with the WT MEFs were presented in Fig 3B. n = 4–6 in each condition. * *P* < 0.05 vs. control of the same genotype. ^#^ *P* < 0.05 vs. WT under the same treatment condition. ^¶^ *P* < 0.05 vs. the same genotype under doxorubicin only condition.

To further validate the notion that different mechanisms mediate the cytotoxic effects induced by doxorubicin and H_2_O_2_, we examined the effects of ROCK1 deletion on caspase activations induced by these two stresses. As previously described [22], ROCK1 deletion efficiently reduced the activations of caspases 3, 8, and 9 by more than 70% in the treatment of 3 μM doxorubicin for 16 h: the maximal levels of caspases 3 and 8 activations were in the range of 4–6 folds in *ROCK1* ^*-/-*^ MEFs (Fig 7B) *versus* 15–25 folds in WT MEFs (Fig 2). Efficient inhibition by ROCK1 deletion on doxorubicin-induced caspase activations could be observed in a dose-dependent study (0.5 to 10 μM) and a time-course study (8 to 24 h) (S6 Fig). Moreover, NAC treatment was more effective in reducing caspase activations in *ROCK1* ^*-/-*^ MEFs compared to WT MEFs: NAC at 2 mM reduced doxorubicin-induced caspase 3 activation by more than 70% in *ROCK1* ^*-/-*^ MEFs (Fig 7B), but less than 30% in WT MEFs (Fig 3B) supporting the additive protection of NAC and ROCK1 deletion. On the other hand, ROCK1 deletion had no apparent effect on H_2_O_2_-induced caspase activation, our data showed similar levels of caspase activations between ROCK1 deletion and WT MEFs after 4 h of H_2_O_2_ treatment with different concentrations from 50 to 200 μM (Fig 7A). These results indicate that H_2_O_2_-induced caspase activation is mediated by ROCK1-independent mechanisms.

**Fig 8:**
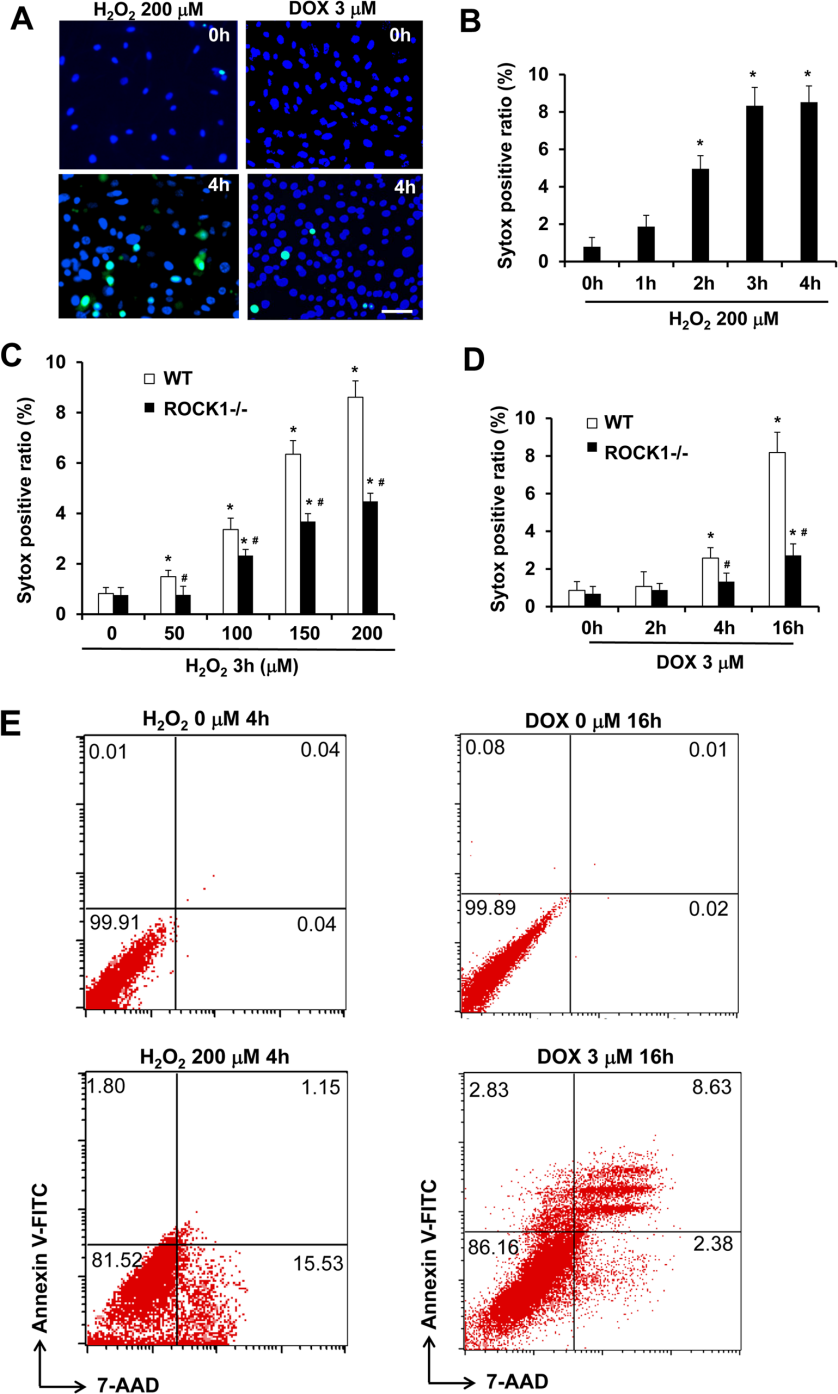
ROCK1 deletion attenuates doxorubicin- and H_2_O_2_-induced necrotic cell death. (A). Representative images of Sytox Green (Green) and Hoechst 33342 staining (blue) of WT MEFs treated with 200 μM of H_**2**_O_**2**_ or 3 μM doxorubicin for 0 or 4 h. Bar, 200 μm. (B-D) Necrotic cells measured by Sytox Green staining in attached WT and/or *ROCK1* ^*-/-*^ MEFs at indicated time points and dosages of H_**2**_O_**2**_ and doxorubicin. The ratio of Sytox Green positive cells was expressed as percentage of attached cells. At least 10,000 cells were analyzed for each condition. * *P* < 0.05 vs. control of the same genotype. ^#^ *P* < 0.05 vs. WT under the same treatment condition. (E) Representative scatter plots of necrosis and apoptosis quantified by FACS analysis after staining with annexin V and 7-AAD in attached WT and ROCK1^-/-^ cells collected after treatment for 4 h with 200 μM of H_**2**_O_**2**_ or 16 h with 3 μM doxorubicin. Viable cells are annexin V−/7-AAD−; annexin V+/7-AAD− cells are in early apoptosis; annexin V+/7-AAD+ cells are in late apoptosis; necrotic cells are annexin V−/7-AAD+. Necrotic cells induced by H_**2**_O_**2**_ are predominantly annexin V negative, whereas the majority of necrotic cells induced doxorubicin are annexin V positive (late apoptosis).

In addition to apoptosis, it has been reported that necrotic cell death also contributes to doxorubicin or H_2_O_2_ cytotoxicity [38,39]. We assessed early necrotic cell death induced by these two stresses within 4 h of treatment by measuring cellular uptake of Sytox Green dye, which is cell membrane impermeable in live cells but permeable in necrotic cells due to compromised cell membrane (Fig 8A). H_2_O_2_ treatment induced necrotic cell death in WT MEFs in a dose and time-dependent manner. The maximal levels of necrotic cell ratio were reached within 4 h (Fig 8B), and the time course of necrotic cell death is similar to that of caspase activation induced by H_2_O_2_ (Fig 2). The rate of necrosis increased when treated with H_2_O_2_ at 50 μM or higher and reached 8–10% at 200 μM H_2_O_2_. This dose-dependent increase of necrosis during the early time window (4 h) suggests that H_2_O_2_ treatment induces primary necrosis. To support this notion, double staining of annexin V and 7-AAD was performed in H_2_O_2_-treated cells followed by flow cytometry analysis, and the results showed that the majority of necrotic cells (7-AAD positive) were annexin V negative, therefore an event independent of apoptosis (Fig 8E). On the other hand, doxorubicin at 3 μM produced a much smaller scale of necrotic cell death, less than 3%, after 4 h, and reached 8–10% at 16 h (Fig 8D). Double staining of annexin V and 7-AAD followed by flow cytometry analysis showed that the majority of necrotic cells (7-AAD positive) were also annexin V positive (late apoptosis), suggesting that necrotic cell death largely occurs secondary to apoptosis after doxorubicin treatment. These results indicate that H_2_O_2_ is a stronger inducer for primary necrotic cell death than doxorubicin in MEFs. These results support that H_2_O_2_ and doxorubicin induce necrotic cell death through different mechanisms in MEFs.

The quantitative analysis of necrosis induced by H_2_O_2_ was performed in parallel with *ROCK1* ^*-/-*^ MEFs (Fig 8C). ROCK1 deficiency showed a significant reduction of necrotic cell ratio compared to WT MEFs after 4 h H_2_O_2_ treatment at 50–200 μM. This reduced necrosis in *ROCK1* ^*-/-*^ MEFs compared to WT MEFs most likely contributes to the moderate reduction in cell detachment and loss of cell viability after treatment with H_2_O_2_ (Fig 5B and 5C). *ROCK1* ^*-/-*^ MEFs also showed reduced numbers of Sytox Green positive cells at both 4 and 16 h after doxorubicin treatment (Fig 8D). The marked reduction of necrosis observed in doxorubicin treated *ROCK1* ^*-/-*^ MEFs compared to WT MEFs at 16 h correlated with the high degree of inhibition of caspase activation by ROCK1 deletion (Fig 7). These results reveal a role for ROCK1 in mediating H_2_O_2_-induced primary necrosis and in doxorubicin-induced necrosis most likely secondary to apoptosis.

## Both NAC and ROCK1 deletion reduce ROS levels in doxorubicin- and H_2_O_2_-treated cells

**Fig 9:**
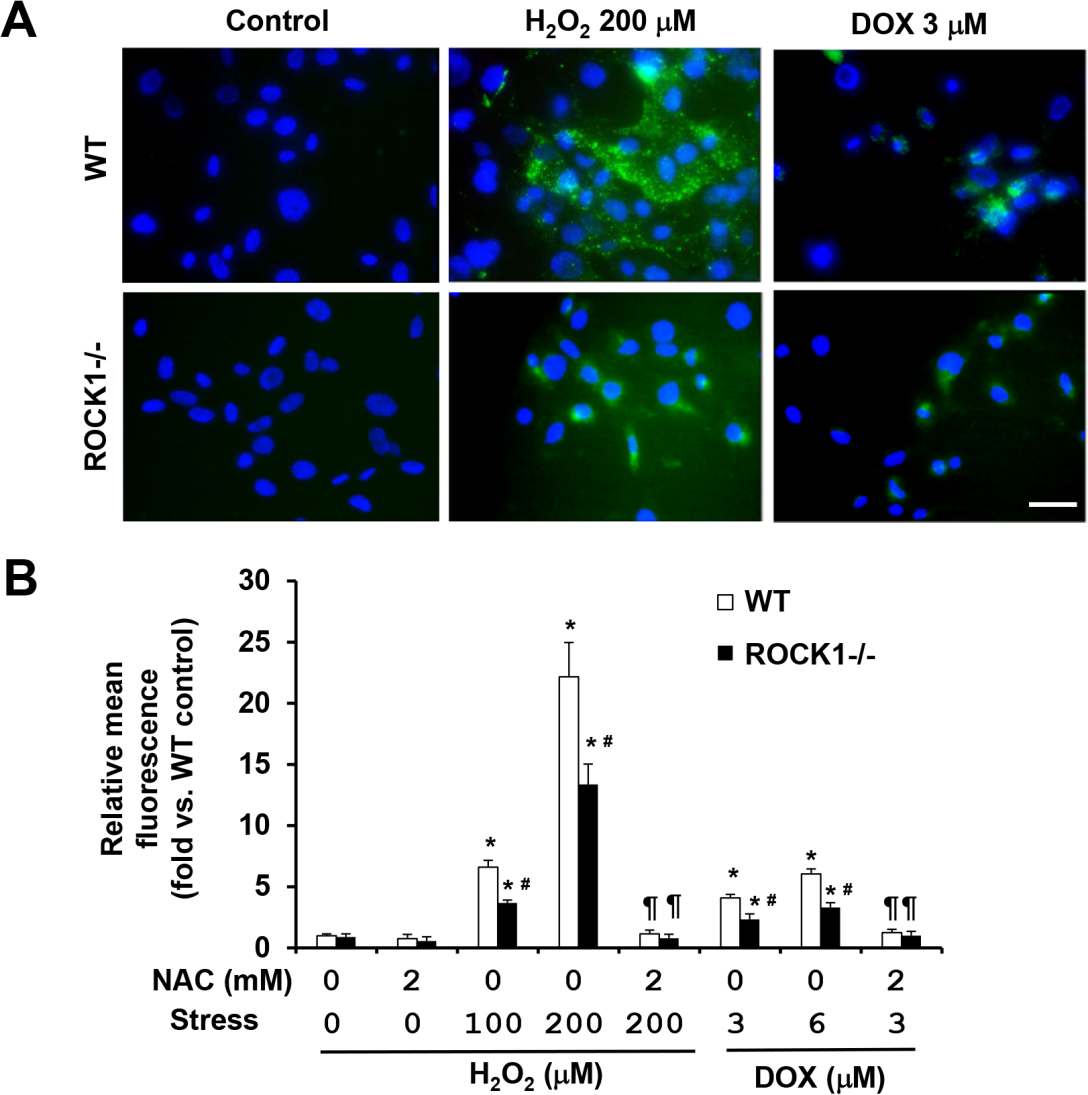
NAC and ROCK1 deletion reduce ROS levels induced by doxorubicin and H_2_O_2_ treatments. (A). Representative images of CM-H2DCFDA staining of WT and *ROCK1* ^*-/-*^ MEFs treated with 200 μM of H_**2**_O_**2**_ or 3 μM doxorubicin, and/or 2 mM NAC for 0 or 4h, and then exposed to 13 μM CM-H2DCFDA. Coverslip was mounted with AntiFade Mountant containing DAPI and imaged immediately. Bar, 50 μm. (B). Quantitative analysis of CM-H2DCFDA staining of WT and *ROCK1* ^*-/-*^ MEFs treated as above followed by the measurement with microplate reader and image analytic system. At least 10,000 cells were analyzed in each condition. Fluorescence levels in WT cells at baseline were arbitrarily set at 1. * *P* < 0.05 vs. control of the same genotype. ^#^ *P* < 0.05 vs. WT under the same treatment condition. ^¶^ *P* < 0.05 vs. the same genotype under doxorubicin or H_**2**_O_**2**_ only condition.

To evaluate effects of NAC and ROCK1 deficiency on oxidative stress induced by doxorubicin or H_2_O_2_ treatment, we measured intracellular ROS levels with chloromethyl derivative of dichlorodihydrofluorescein diacetate (CM-H2DCFDA), an oxidant-sensitive dye (Fig 9A). The treatment with doxorubicin or H_2_O_2_ for 3 h increased ROS levels in WT MEFs in a dose-dependent manner. Interestingly, 3 μM doxorubicin treatment produced much smaller increases of ROS levels (3 to 4-fold) compared to those produced by 200 μM H_2_O_2_ treatment (20 to 25-fold) (Fig 9B), correlating with lower necrosis induced by doxorubicin compared those induced by H_2_O_2_ treatment for 3 to 4 h (Fig 8). Co-treatment with 2 mM of NAC completely suppressed the increased ROS levels in both doxorubicin and H_2_O_2_ treated cells (Fig 9B), supporting that increased intracellular ROS levels contribute to necrosis. In addition, ROCK1 deficiency showed a significant reduction of ROS levels compared to WT MEFs after doxorubicin or H_2_O_2_ treatment (Fig 9B). This reduced ROS levels in *ROCK1* ^*-/-*^ MEFs compared to WT MEFs most likely contributes to the reduced necrosis induced by H_2_O_2_ or by doxorubicin at early time points (Fig 8C and 8D).

## Discussion

**Fig 10:**
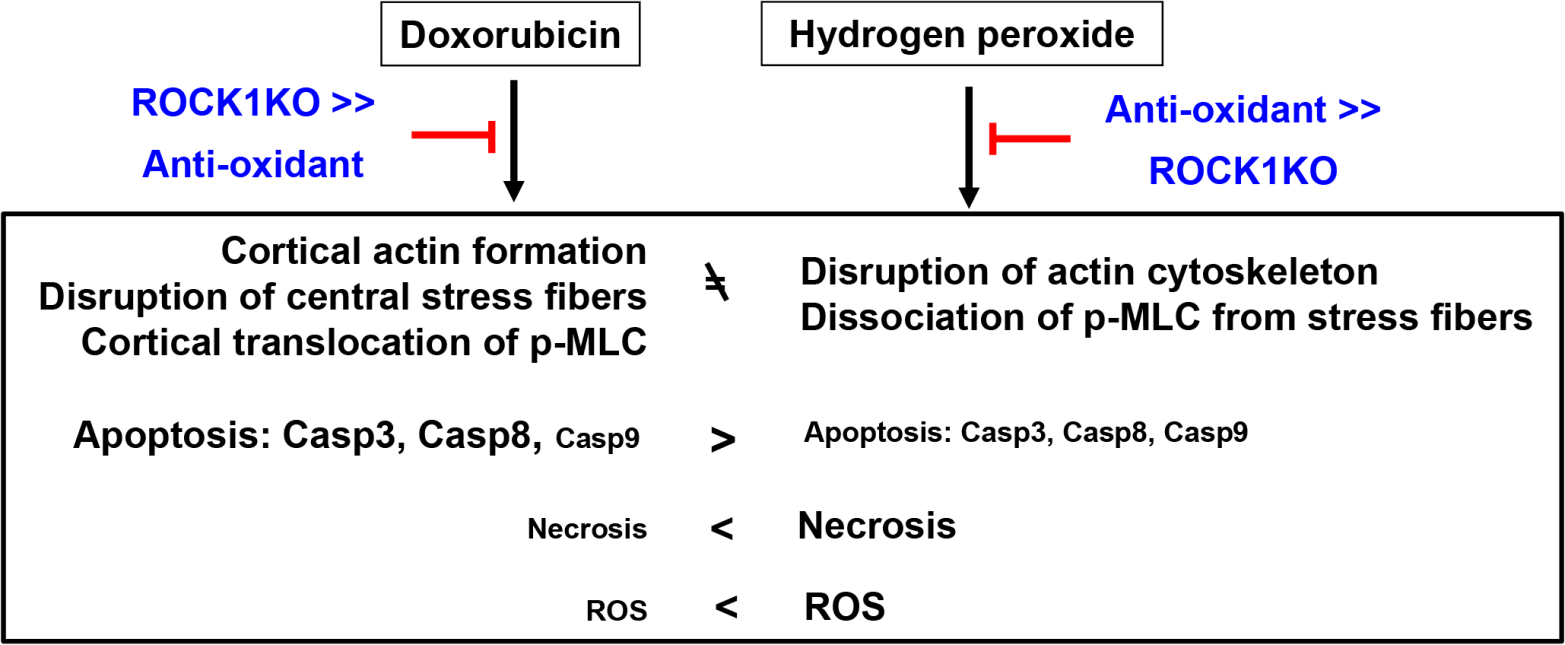
Schematic summary. The diagram summarizes the effects of antioxidant (NAC) and ROCK1 deficiency in opposing apoptosis, necrosis, ROS production and actin cytoskeleton alterations induced by H_**2**_O_**2**_ and doxorubicin. Antioxidant treatment shows a stronger protection than ROCK1 deletion against H_**2**_O_**2**_-induced cytotoxic effects while ROCK1 deletion shows a stronger protection than antioxidant treatment against doxorubicin-induced cytotoxicity. These results support the notion that doxorubicin induces apoptosis, necrosis, and actin cytoskeleton alterations predominantly through a ROS-independent and ROCK1-dependent mechanisms. Additional results supporting this concept include different temporal patterns and magnitudes of caspase activations and necrotic cell death, different levels of ROS production and different alteration of actin cytoskeleton induced by H_**2**_O_**2**_ compared to doxorubicin.

We recently reported that ROCK1 deficiency in MEFs has superior anti-apoptotic and pro-survival effects compared to anti-oxidants against doxorubicin. However, this drug-induced oxidative stress is the most widely accepted underlying mechanism attributable to its cytotoxicity to normal cells, especially to cardiomyocytes. The present study investigates the mechanistic differences between the cytotoxic effects of doxorubicin compared to H_2_O_2_. The approaches consist of comparing the cytotoxic effects induced by doxorubicin *versus* those induced by H_2_O_2_ in WT and *ROCK1* ^*-/-*^ MEFs, and in the presence or absence of the antioxidant NAC. Although both H_2_O_2_ and doxorubicin induce significant cytotoxicity in WT MEFs including apoptosis, necrosis, cell detachment, actin cytoskeleton alterations and excessive ROS production, our analyses reveal that these cytotoxic events clearly present with different characteristics (Fig 10). The present study reveals that the drug-induced actin cytoskeleton remodeling (ROCK1-dependent) plays a more important role than the drug-induced oxidative stress in mediating doxorubicin cytotoxicity in MEFs.

The present study reveals striking differences between H_2_O_2_- and doxorubicin-induced caspase activation: H_2_O_2_-induced activations of caspases 3, 8, and 9 occur earlier (within 4 h) and with much lower maximal levels (2 to 4-fold) compared to those induced by doxorubicin (8 to 24 h and 15 to 25-fold activation for caspase 3 and 8). These differences in caspase activation are associated with different actin cytoskeleton remodeling induced by H_2_O_2_ and doxorubicin: H_2_O_2_ induces disruption of stress fibers accompanied by dissociation of p-MLC from stress fibers to the cytoplasm, while only central stress fibers are disrupted by doxorubicin accompanied by increased cortical stress fibers and cortical translocation of p-MLC from central stress fibers. Although the antioxidant NAC suppresses both H_2_O_2_- and doxorubicin-induced ROS production and also H_2_O_2_-induced caspase activation and cytoskeleton remodeling, it has limited effects on doxorubicin-induced caspase activation and cytoskeleton remodeling. Importantly, ROCK1 deficiency has more potent inhibition on doxorubicin-induced caspase activation and cytoskeleton remodeling than those induced by H_2_O_2_. These results support the notion that doxorubicin induces actin cytoskeleton alterations, impairs cell adhesion, and increases caspase activations largely through ROS-independent and ROCK1-dependent mechanisms.

Although many studies have suggested that antioxidants have protective effects on chemotherapy-induced cardiotoxicity, clinical trials of antioxidant therapy showed unsatisfactory benefits [18,19]; the reasons for it are still undetermined. Our results from the current study support a central role for ROCK1 in promoting p-MLC translocation to cortical stress fibers resulting in excessive cortical actomyosin contraction induced by doxorubicin. Two pieces of evidence from the current study indicate that this ROCK1-mediated translocation of p-MLC to the cortical stress fibers occurs independently of oxidative stress: 1) NAC treatment has minor effects on doxorubicin-induced cortical stress fiber formation; 2) H_2_O_2_ treatment does not induce cortical translocation of p-MLC, but instead, the treatment removes p-MLC from all stress fibers. In line with our previous report, the current study also supports that ROCK1 deletion and antioxidant have additive effects in reducing apoptosis, cell detachment and loss of cell viability induced by doxorubicin [34]. It would be of interest to explore the mechanisms through which doxorubicin treatment triggers ROCK1-mediated cortical translocation of p-MLC. Although MEFs are not primary targets of doxorubicin cardiotoxicity, the advantage of using these cells in the present mechanistic study is that actin cytoskeleton (stress fibers) can be easily visualized, therefore facilitating the investigation of their contribution to caspase activation, cell detachment and the loss of cell viability induced by cytotoxic stresses. It would be of interest to validate these mechanisms in other cell types such as cardiomyocytes, endothelial cells, and progenitor cells which are primary targets for doxorubicin cardiotoxicity [35,40–43].

In contrast to the strong protection on doxorubicin-induced cytoskeleton remodeling, the current study reveals that ROCK1 deletion has no apparent protection on stress fiber disruption induced by H_2_O_2_, which causes dislocation of p-MLC from all stress fibers including cortical stress fibers. The observation of the deletion of ROCK1 cannot prevent H_2_O_2_-induced dislocation of p-MLC from stress fibers is consistent with the established primary role of ROCK1 in promoting p-MLC association with stress fibers. This reduced actomyosin contraction due to dislocation of p-MLC from stress fibers is a key factor in the disruption of stress fibers by H_2_O_2_. In agreement with this idea, we observed that the inhibition of actomyosin contraction by blebbistatin, a direct inhibitor of myosin II ATPase, further increases H_2_O_2_-induced cell detachment while it decreases doxorubicin-induced cell detachment.

In association with the lack of protection on actin cytoskeleton in H_2_O_2_ treatment, ROCK1 deletion does not inhibit caspase activation, and has only moderate inhibition on cell detachment and the loss of cell viability. The moderate protective effects of ROCK1 deletion on H_2_O_2_-induced loss of cell viability are most likely through inhibition of necrosis. We observed that the anti-necrotic action of ROCK1 deletion correlates with its effect of partially reducing the ROS levels. In addition, the higher levels of primary necrosis-induced by H_2_O_2_ correlate with the higher levels of intracellular ROS levels compared to those-induced by doxorubicin in MEFs, supporting that oxidative stress is a major contributor to the primary necrosis induced by H_2_O_2_ or doxorubicin. We previously observed that ROCK1 deletion reduces doxorubicin-induced ROS production through inhibiting NADPH oxidase activation [34]. Future studies are needed to determine the mechanisms through which ROCK1 deletion reduces H_2_O_2_-induced excessive ROS. It is worth noting that ROS has been previously reported to induce RhoA/ROCK activation [44,45], supporting that ROCK1 is downstream of H_2_O_2_. The current study indicates that ROCK1 can also act upstream of H_2_O_2_ and be involved in the regulation of ROS production.

In summary, this study has shown that oxidative stress and doxorubicin produce actin cytoskeleton alteration, apoptosis, and necrosis with clearly different features in MEFs. Antioxidant treatment and ROCK1 deletion have distinct protective effects for these two stresses: an antioxidant is more effective on opposing oxidative stress cytotoxicity, while ROCK1 deletion is more effective on resisting doxorubicin cytotoxicity (Fig 10). These results support the notion that doxorubicin induces cortical stress fiber formation, impairs cell adhesion, increases caspase activations largely through oxidative stress-independent and ROCK1-dependent mechanisms. These observations also highlight the importance of actin cytoskeleton remodeling in stress responses: an increase in cortical actomyosin contraction (ROCK1-dependent) is linked with impaired cell adhesion, high level of apoptosis induction and low level of primary necrosis induction in response to doxorubicin; a decrease in actomyosin contraction is linked to impaired cell adhesion, low level of apoptosis induction, and high level of primary necrosis induction in response to H_2_O_2_ treatment. Our studies support the need to investigate actin cytoskeleton changes in response to other cytotoxic stresses (i.e. other anti-cancer drugs and other environmental stresses) and the impact of the actin cytoskeleton changes on cytotoxic mechanisms such as apoptosis and necrosis.

## Materials and Methods

All animal experiments were conducted in accordance with the National Institutes of Health “Guide for the Care and Use of Laboratory Animals” (NIH Publication No. 85–23, revised 1996) and were approved by the Institutional Animal Care and Use Committee at Indiana University School of Medicine.

A detailed description of the materials and methods used in the present study can be found in S1 File (Supplemental Materials and Methods).

### Cell culture and drug treatment

WT and ROCK1-deficient MEF cells were prepared from WT and *ROCK1* ^-/-^ embryos as previously described [22]. Cells were cultured as previously described [22], and were treated at ~90% confluence with hydrogen peroxide, doxorubicin, and NAC (Sigma-Aldrich) at indicated times and dosages.

### Cell viability, apoptosis and necrosis and detachment assays

Following the treatment of desired drugs at indicated concentrations, cell viability measured by MTT assay, cell detachment assay by counting the detached (floating) and attached (collected by trypsinization) cells were performed as previously described [22]. Flow cytometry was used for quantitative analysis of apoptotic and necrotic cells as previously described [22], using a FITC Annexin V Apoptosis Detection Kit with 7-AAD (BioLegend).

Cytation 3 (BioTek Instruments), a cell-based multi-mode microplate reader and image analysis system, was used for quantitative analysis of Sytox Green (Life Technologies) uptake by necrotic cells. The ratio of necrotic cells with compromised cellular membranes can be measured by fluorescence imaging of 24-well plates combined with image-based quantitative analyses of green nuclei (Sytox Green positive cells) and blue nuclei (Hoechst 33342 staining for total cells). The samples were prepared in duplicate and at least 5,000 cells were analyzed for each well. At least five independent experiments were analyzed.

### Fluorescence imaging

Rhodamine-phalloidin staining of F-actin and immunostaining of p-MLC were followed by fluorescence microscopy detection as previously described [22]. ROS detection was performed in live cells with CM-H2DCFDA (C-6827, Life Technologies) as previously described [34].

### Protein analysis

Following treatment with desired drugs, attached cells were collected for further analyses. Protein samples were prepared from cell lysates, and subsequently analyzed by Western blot as previously described [22].

### Statistical analysis

We analyzed all data by using Student’s *t*-test or ANOVA as appropriate. A *P* < 0.05 was considered as significant. Data are presented as mean ± SE.

## Supporting Information

S1 Fig Dose-dependent activation of caspases by H2O2 and doxorubicin. (DOC)

S2 Fig Time-dependent activation of caspases by doxorubicin. (DOC)

S3 Fig NAC shows limited effect on doxorubicin-induced caspase activation. (DOC)

S4 Fig H2O2 induces reduction of stress fibers and cytosolic translocation of p-MLC. (DOC)

S5 Fig Inhibition of actomyosin contraction by blebbistatin increases H2O2-induced cell detachment, but decreases doxorubicin-induced cell detachment. (DOC)

S6 Fig ROCK1 deletion has strong inhibition on doxorubicin-induced caspase activation. (DOC)

S1 File Supplemental Materials and Methods. (DOC)
