# Supplementary material for: Dissecting the Mechanisms of Doxorubicin and Oxidative Stress-Induced Cytotoxicity: The Involvement of Actin Cytoskeleton and ROCK1
Source: PLoS One. 2015 Jul 2;10(7):e0131763. doi: 10.1371/journal.pone.0131763 (PMC4489912; doi:10.1371/journal.pone.0131763)
Supplement: S1 Fig — (DOC) [file pone.0131763.s001.doc]

# (2014) ROCK1 deficiency enhances protective effects of antioxidants against apoptosis and cell detachment. PLoS One 9: e90758.

**S1 Fig. Dose-dependent activation of caspases by H_2_O_2_ and doxorubicin.** Representative image of Western blot of cleaved caspases 3, 8, and 9 (top) and quantitative analysis of Western blot of cleaved caspase 3 (bottom) in cell lysates from attached WT MEFs treated with H_2_O_2_ (A) or doxorubicin (B) at indicated concentrations for 16 h. Equal amount of proteins were loaded. n = 4-6 in each treatment condition. Activation of caspases by H_2_O_2_ reaches maximal levels at 100-200 µM. Activation of caspases by doxorubicin reaches maximal levels at 3-10 µM. * *P* < 0.05 vs. control.
