# Supplementary material for: Dissecting the Mechanisms of Doxorubicin and Oxidative Stress-Induced Cytotoxicity: The Involvement of Actin Cytoskeleton and ROCK1
Source: PLoS One. 2015 Jul 2;10(7):e0131763. doi: 10.1371/journal.pone.0131763 (PMC4489912; doi:10.1371/journal.pone.0131763)
Supplement: S2 Fig — (DOC) [file pone.0131763.s002.doc]

# (2014) ROCK1 deficiency enhances protective effects of antioxidants against apoptosis and cell detachment. PLoS One 9: e90758.

**S2 Fig. Time-dependent activation of caspases by doxorubicin.**

Representative image of Western blot of cleaved caspases 3, 8, and 9 (left) and quantitative analysis of Western blot of cleaved caspase 3 (right) in cell lysates from attached WT MEFs treated with 3 µM doxorubicin at indicated time points. Equal amount of proteins were loaded. n = 4-6 in each treatment condition. Activation of caspases by doxorubicin reaches a plateau at 16-24 h. * *P* < 0.05 vs. control.
