# Supplementary material for: Dissecting the Mechanisms of Doxorubicin and Oxidative Stress-Induced Cytotoxicity: The Involvement of Actin Cytoskeleton and ROCK1
Source: PLoS One. 2015 Jul 2;10(7):e0131763. doi: 10.1371/journal.pone.0131763 (PMC4489912; doi:10.1371/journal.pone.0131763)
Supplement: S3 Fig — (DOC) [file pone.0131763.s003.doc]

# (2014) ROCK1 deficiency enhances protective effects of antioxidants against apoptosis and cell detachment. PLoS One 9: e90758.

**S3 Fig. NAC shows limited effect on doxorubicin-induced caspase activation.** (A**)** Representative image (top) and quantitative analysis (bottom) of Western blot of cleaved caspase 3 in cell lysates from attached WT MEFs treated for 16 h with 3 μM doxorubicin and different concentrations of NAC as indicated. (B**)** Representative image (top) and quantitative analysis (bottom) of Western blot of cleaved caspase 3 in cell lysates from attached WT MEFs treated for 16 h with 2 mM NAC and different concentration of doxorubicin as indicated. Equal amount of proteins were loaded. n = 4-6 in each treatment condition. * *P* < 0.05 vs. control. ^#^*P* < 0.05 vs. doxorubicin only condition.
