# Supplementary material for: Dissecting the Mechanisms of Doxorubicin and Oxidative Stress-Induced Cytotoxicity: The Involvement of Actin Cytoskeleton and ROCK1
Source: PLoS One. 2015 Jul 2;10(7):e0131763. doi: 10.1371/journal.pone.0131763 (PMC4489912; doi:10.1371/journal.pone.0131763)
Supplement: S4 Fig — (DOC) [file pone.0131763.s004.doc]

# (2014) ROCK1 deficiency enhances protective effects of antioxidants against apoptosis and cell detachment. PLoS One 9: e90758.

**S4 Fig. H_2_O_2_ induces reduction of stress fibers and cytosolic translocation of p-MLC.**

Representative images of rhodamine phalloidin staining of F-actin (red), p-MLC staining (green), and DAPI staining (blue) in WT MEFs treated with 200 µM of H_2_O_2_ for 4, or 8 h. Bar, 50 µm. The staining of WT MEFs in control shows co-localization of p-MLC with F-actin. WT MEFs treated with H_2_O_2_ show diffused cytoplasmic p-MLC staining: heterogeneous pattern at 4 h with some cells showing high and some showing low levels of staining; homogenous pattern at 8 h with all cells showing low levels of staining.
