# Supplementary material for: Dissecting the Mechanisms of Doxorubicin and Oxidative Stress-Induced Cytotoxicity: The Involvement of Actin Cytoskeleton and ROCK1
Source: PLoS One. 2015 Jul 2;10(7):e0131763. doi: 10.1371/journal.pone.0131763 (PMC4489912; doi:10.1371/journal.pone.0131763)
Supplement: S5 Fig — (DOC) [file pone.0131763.s005.doc]

# (2014) ROCK1 deficiency enhances protective effects of antioxidants against apoptosis and cell detachment. PLoS One 9: e90758.

**S5 Fig. Inhibition of actomyosin contraction by blebbistatin increases H_2_O_2_-induced cell detachment, but decreases doxorubicin-induced cell detachment.**

Both floating and attached WT MEFs were collected after16 h treatment with 200 μM H_2_O_2_ or 3 μM doxorubicin in the presence or absence of 1 μM blebbistatin. Floating cell ratio was expressed as percentage of total cells (floating plus attached cells) in each treatment condition. At least three independent experiments were analyzed. ***** *P* < 0.05 vs. control of the same genotype. ^#^*P* < 0.05 vs. H_2_O_2_ or doxorubicin only condition.
