# Supplementary material for: Dissecting the Mechanisms of Doxorubicin and Oxidative Stress-Induced Cytotoxicity: The Involvement of Actin Cytoskeleton and ROCK1
Source: PLoS One. 2015 Jul 2;10(7):e0131763. doi: 10.1371/journal.pone.0131763 (PMC4489912; doi:10.1371/journal.pone.0131763)
Supplement: S6 Fig — (DOC) [file pone.0131763.s006.doc]

# (2014) ROCK1 deficiency enhances protective effects of antioxidants against apoptosis and cell detachment. PLoS One 9: e90758.

**S6 Fig. ROCK1 deletion has strong inhibition on doxorubicin-induced caspase activation.**

Representative image of Western blot of cleaved caspases 3, full length and cleaved ROCK1 in cell lysates from attached WT and *ROCK1^-/-^* MEFs treated for 16 h with different concentrations of doxorubicin as indicated (A) or with 3 μM doxorubicin for 2 to 8 h (B), or with 3 μM doxorubicin for 24 h (C). ROCK1 deficient MEFs show reduced activation of caspase 3 in indicated examines.
