# Supplementary material for: Dissecting the Mechanisms of Doxorubicin and Oxidative Stress-Induced Cytotoxicity: The Involvement of Actin Cytoskeleton and ROCK1
Source: PLoS One. 2015 Jul 2;10(7):e0131763. doi: 10.1371/journal.pone.0131763 (PMC4489912; doi:10.1371/journal.pone.0131763)
Supplement: S1 File — (DOC) [file pone.0131763.s007.doc]

# Supplemental Materials and Methods

**Dissecting the mechanisms of doxorubicin and oxidative stress-induced cytotoxicity: the involvement of actin cytoskeleton and ROCK1**

Lei Wei*, Michelle Surma, Gina Gough, Stephanie Shi, Nathan Lambert-Cheatham, Jiang Chang, Jianjian Shi*

## ^*^To whom correspondence should be addressed. E-mail: [**jishi@iu.edu**](mailto:jishi@iu.edu) (J.S.) or [**lewei@iu.edu**](mailto:lewei@iu.edu) (L.W.)

## Supplemental Materials and Methods

**Materials and Methods**

**Cell culture and treatments.** WT and ROCK1-deficient MEF cells were prepared from WT and *ROCK1*^-/-^ embryos respectively as previously described . All animal experiments were conducted in accordance with the National Institutes of Health “Guide for the Care and Use of Laboratory Animals” (NIH Publication No. 85-23, revised 1996) and were approved by the Institutional Animal Care and Use Committee at Indiana University School of Medicine. Cells were cultured in Dulbecco’s modified Eagle’s medium (DMEM; Life Technologies) supplemented with 10% fetal bovine serum (FBS; Atlanta Biologicals) and penicillin-streptomycin in a humidified incubator with 5% CO_2_ at 37°C. Cells at 85-90% confluence were treated with hydrogen peroxide, doxorubicin, and NAC (Sigma-Aldrich) at indicated times and dosages.

**Cell viability and detachment assays.** For cell viability assay, following treatment with desired drugs at indicated concentrations and time points, MTT assay was performed as previously described . At least three independent experiments were analyzed.

The ratio of necrotic cells (with compromised cellular membranes) to normal cells is measured with Cytation 3 (BioTek) cell-based multi-mode microplate reader and image analytic system. SYTOX Green (Life Technology) stain can easily penetrate cells with compromised plasma membranes but does not cross the membranes of live cells making it a useful indicator of dead cells; upon the binding of nucleic acids of dead cells with SYTOX Green, bright green fluorescent positive cells can be viewed under a fluorescence microscope or with a Cytation 3 equipped with a standard fluorescein filter set (approximate fluorescence excitation/emission maxima at 504/523 nm). After finishing treatment at indicated condition, MEFs were brief washed with pre-warmed HBSS, and incubated with SYTOX Green nucleic acid stain for 15 min, followed by removing the dye and replacing with Hoechst 33342 (Life Technology) stain solution for counting total cell population.

The ratio of dead/live cells is measured by analyzing fluorescence images obtained from of 24-well plates, all test cell samples were set in duplication and at least 5,000 cells were analyzed for each well. More than five independent experiments were performed.

Flow cytometry was used for quantitative analysis of apoptotic and necrotic cells as previously described . Apoptotic and dead cells were determined by staining with FITC- annexin V and 7-AAD (FITC Annexin V Apoptosis Detection Kit with 7-AAD, BioLegend), respectively. The samples were prepared in triplicate, and each cell preparation was analyzed twice.

For the cell detachment assay, cells were cultured in DMEM supplemented with 10% FBS to reach 90% confluence, followed by the treatment of desired drugs at indicated concentrations for 16 h. Detached cells in culture medium (floating cells) were collected and counted with a hemocytometer. The attached cells were harvested by trypsinization for counting. At least three independent experiments were analyzed in each treatment condition.

**Fluorescence imaging.** Fluorescent phalloidin staining of F-actin and immunofluorescence staining of p-MLC were performed as previously described . The fluorescent images were taken with Leica DM5500B microscope (objectives: HCX PL FUOTAR 20.0 x 0.50, HCX PL FUOTAR 40 x 0.75) equipped with a DFC300FXR2 camera, and images were analyzed with the Leica AF6000 software.

ROS detection by fluorescence imaging was performed as previously described with minor modification. Briefly, we detected ROS production in live cells with CM-H2DCFDA (C-6827, Life Technologies) as a probe, which is membrane permeable for live cells, and has been used as a reliable fluorogenic marker of ROS in numerous cell biology studies. MEF cells were seeded in sterile chamber slide (Lab-Tek) for overnight. After finishing the treatment at the indicated condition, CM-H2DCFDA was added to fresh culture medium at a final concentration of 13 μM, and incubated for 30 min in dark at 37 °C. Cells were then washed once with pre-warmed phosphate-buffered saline (DPBS), followed by replacing ROS probe with FluoroBrite DMEM and continuing incubation for 30 min. Slides were mounted with AntiFade Mountant with DAPI (Life Technology). The oxidative stressed and non-stressed cells were able to be distinguished under fluorescence microscopy or multi-mode microplate reader and image analytic system.

**Protein analysis.** Following treatment with desired drugs, attached cells were collected and analyzed for caspase activation by Western blot analysis as previously described . The blots were then probed with primary antibodies to ROCK1 (GTX61382) from GeneTex, ROCK2 (sc-5561) from Santa Cruz Biotechnology, cleaved caspase-3 (#9661), cleaved caspase-9 (#9509), cleaved caspase-8 (#9429) from Cell Signaling. After blotting with corresponding secondary antibodies conjugated with horseradish peroxidase, the membranes were developed with ECL or SuperSignal West Pico Chemiluminescent Substrate (Thermo Scientific), and the blots were visualized by using a Fujifilm LAS-4000 Imager. All blots were normalized to GAPDH (MA5-15738, Thermo Scientific) or to actin (sc-1616; Santa Cruz Biotechnology).

## Statistical analysis. Data are reported as mean ± SE. Comparisons between groups were analyzed by Student’s *t*-test or ANOVA as appropriate, with *P* < 0.05 considered as significant.

## References

1. Shi J, Wu X, Surma M, Vemula S, Zhang L, Yang Y, et al. (2013) Distinct roles for ROCK1 and ROCK2 in the regulation of cell detachment. Cell Death Dis 4: e483.

2. Surma M, Handy C, Chang J, Kapur R, Wei L, Shi J. (2014) ROCK1 deficiency enhances protective effects of antioxidants against apoptosis and cell detachment. PLoS One 9: e90758.
